# The intra-neuroendoscopic technique (INET): a modified minimally invasive technique for evacuation of brain parenchyma hematomas

**DOI:** 10.1186/s13017-019-0239-0

**Published:** 2019-05-06

**Authors:** Yujuan Zhang, Ai-Jun Shan, Yu-Ping Peng, Pengfei Lei, Jianzhong Xu, Xianliang Zhong, Bo Du

**Affiliations:** 1 Department of Ultrasound, Shenzhen People’s Hospital, The Second Clinical Medical College of Jinan University, The First Affiliated Hospital of Southern University of Science and Technology, Shenzhen, 518020 Guangdong China; 2 Department of Emergency, Shenzhen People’s Hospital, The Second Clinical Medical College of Jinan University, The First Affiliated Hospital of Southern University of Science and Technology, Shenzhen, 518020 Guangdong China; 3 Department of Neurosurgery, Nanfang Hospital, Southern Medical University, Guangzhou, China

**Keywords:** Intra-neuroendoscopy technique (INET), Transparent sheath, Brain parenchyma hematoma, Minimally invasive surgery, Outcome

## Abstract

**Background:**

Minimally invasive endoscopic hematoma evacuation is widely used in the treatment of intracerebral hemorrhage. However, this technique still has room for improvement. The intra-neuroendoscopic technique (INET) is a modified minimally invasive technique, and we report its safety and efficacy in evacuating brain parenchyma hematomas by comparing it with cranial puncture and drainage operation (CPDO).

**Methods:**

The frontal, temporal, or occipital approaches were used according to the site of bleeding. The preoperative and postoperative hematoma volumes, Glasgow Coma Scale (GCS) score, Cerebral State Index (CSI), hematoma evacuation rate, operation time, complications, and 30-day mortality and Glasgow Outcome Scale (GOS) were retrospectively compared between the two groups.

**Results:**

A total of 98 patients were enrolled. The evacuation rate (84 ± 7.1% versus 51.0 ± 8.4%, *p* = 0.00), 7-day GCS (11.8 ± 1.2 versus 10.4 ± 1.5, *p* = 0.01), and CSI (87.1 ± 8.7 versus 80.6 ± 10.2, *p* = 0.02) were higher, and the 30-day mortality rate (1.9% versus 15.6%, *p* = 0.036) was lower in the INET group. However, the operation time was longer in the INET group than in the control group (65.2 ± 12.5 min versus 45.6 ± 10.9 min, *p* = 0.000). Multivariable logistic regression showed that a good medium-term outcome (GOS scores 4–5) was significantly associated with INET (odds ratio (OR) 3.514, 95% confidence interval (CI) 1.463–8.440, *p* = 0.005), age under 65 years (OR 1.402, 95% CI, 1.041–1.888, *p* = 0.026), and hematoma volume less than 50 ml (OR 1.974, 95% CI 1.302–2.993, *p* = 0.001).

**Conclusions:**

INET surgery for brain parenchyma hematoma evacuation is a safe and efficient modified technique. This technique is minimally invasive, has less complications, and may be helpful in providing optimal outcomes for selected patients.

**Trial registration:**

ClinicalTrials.gov, NCT02515903. Registered on 5 August 2015.

**Electronic supplementary material:**

The online version of this article (10.1186/s13017-019-0239-0) contains supplementary material, which is available to authorized users.

## Introduction

Spontaneous intracerebral hemorrhage (ICH) is one of the leading causes of mortality and morbidity [1], with approximately 40% 1-month mortality rates [2–4]. Furthermore, over 60% of surviving patients are reported to have moderate to severe disability [5]. Hypertensive hemorrhages are the most important risk factor for ICH, as well as other causes of bleeding, including cerebral vascular malformations and cerebral aneurysms. The site of brain parenchyma hemorrhage is mainly located in the basal ganglia area (including the putamen and thalamus), frontal lobe, and occipital lobe. ICH requires a high level of medical care, making it one of the most costly neurological diagnoses [6]. Given the requirement for better treatment options for this common and devastating condition, treatment strategies and methods remain controversial despite extensive research on ICH treatment.

Theoretically, surgical evacuation of hematomas could reduce many secondary injuries associated with ICH, such as those caused by its mass effects and the hemotoxicity of the blood breakdown products. However, the traditional craniotomy hematoma evacuation surgery did not show superiority in some randomized trials [7]. The long surgical duration and great damage to viable tissue in traditional craniotomy hematoma evacuation may offset the beneficial effects of the procedure, especially in the case of a deep-seated brain parenchyma hematoma [8, 9]. Therefore, minimally invasive surgical techniques that could shorten the surgical duration are more acceptable and are being investigated. Recently, the following two minimally invasive approaches have mainly been used: the cranial puncture and drainage operation (CPDO) and the endoscopic hematoma evacuation operation (EHEO). The CPDO was demonstrated to have beneficial effects for spontaneous intracerebral hemorrhage patients in a small randomized controlled trial and a meta-analysis [10, 11]. In recent years, with improvements in endoscopic technology and equipment, the EHEO has been regarded as a more promising method to treat ICH [12–18]. Compared with the CPDO, the endoscopic approach can achieve immediate hematoma evacuation, reducing the possibility of cumulative secondary injuries [9, 19]. In addition, the endoscopic approach has an impressive short-term outcome, low re-hemorrhaging rate, and less surgery-related morbidity [12, 13, 15, 16, 18, 20–22].

Reviewing these reported case series, we noted the following. (1) The sheath is different. Some groups used a metal sheath, whereas others used a transparent sheath. The latter is superior to the former in many ways. However, its diameter is approximately 20 mm, and the surgery requires the removal of a 3-cm diameter bone flap, necessitating allogeneic material fixation after the operation [23]. (2) During the process of implanting the transparent sheath, most of the hematoma is not visible. (3) Endoscopic surgery is mainly carried out in an air environment, and an operation environment with continuous fluid flush is not easy to achieve. The surgical operation is performed in the transparent sheath but outside of the endoscope, whose only role is to provide light. (4) It is still challenging to remove hard hematomas.

In our hospital, we started performing endoscopic hematoma evacuation surgery in September 2015. We have developed a transparent sheath (Chinese Patent No. ZL 200820046232.0, State Intellectual Property Office of P.R. China, website http://cpquery.sipo.gov.cn) for large-channel neuroendoscope, which achieved a visible puncture. We also developed a hematoma smashing suction apparatus (Chinese Patent No. ZL 201520248717.8, State Intellectual Property Office of P.R. China, website http://cpquery.sipo.gov.cn) to solve the problem of removing hard hematomas. We designed a non-randomized concurrent control trial, where the control was a CPDO group. The clinical efficacy and safety of the new method were discussed based on this study.

## Material and methods

### Ethics statement

The Medical Science Ethics Committee of the Southern Medical University approved this study (NFEC-2015-034). Each patient or an appropriate family member provided informed written consent to obtain clinical materials.

### Study population

From September 2015 to May 2018, a total of 98 patients met the inclusion criteria for the study, with 53 patients in the intra-neuroendoscopic technique (INET) treatment group and 45 patients in the control group. All patients were enrolled from the neurosurgery department of Nanfang Hospital and the emergency center of Shenzhen People’s Hospital. The baseline data of the two groups are shown in Table 1. All patients underwent brain computed tomography (CT) to determine the diagnosis, and magnetic resonance angiography (MRA), CT angiography, or digital subtraction angiography (DSA) was performed to rule out patients with an arteriovenous malformation or an aneurysm. The Tada formula [24] was used to calculate the hematoma volume.

**Table 1:** Comparison of baseline indicators between the INET and control groups

Baseline indicators	INET group (*n* = 53)	Control group (*n* = 45)	*p* value
Age ≤ 65 years	27 (50.9%)	33 (73.3%)	0.023
Preoperative GCS	8.9 ± 1.4	8.3 ± 1.6	0.432
Preoperative hematoma volumes ≤ 50 ml	40 (75.5%)	25 (55.6%)	0.038
Preoperative CSI	76.1 ± 7.9	71.9 ± 7.4	0.337
Hemorrhage sites			
Basal ganglia	40 (75.4%)	33 (73.3%)	0.809
Frontal lobe	6 (11.3%)	4 (8.9%)	0.951
Parietal lobe	3 (5.7%)	3 (6.7%)	0.829
Occipital lobe	4 (7.5%)	5 (11.1%)	0.797

### Inclusion and exclusion criteria

#### Inclusion criteria

The inclusion criteria are as follows: (1) Diagnosis of spontaneous hemorrhage in the parenchyma of the brain on CT scan. (2) Hemorrhage volume was greater than 25 ml, and no brain hernia was formed. (3) Age range was 40–75 years. (4) Hemorrhagic duration (from stroke onset to hospital) was less than 72 h. (5) Informed consent was provided from patients and/or their relatives.

#### Exclusion criteria

The exclusion criteria are as follows: (1) Disturbance in blood coagulation, such as thrombocytopenia or hepatitis; (2) intracranial or general infection; (3) co-existence of severe heart, liver, kidney, or lung disease or functional failure; (4) a previous history of stroke with neurological deficits; (5) under anticoagulation therapy; and (6) hemorrhage caused by brain injury, intracranial aneurism or cerebral arteriovenous malformations.

### Surgical techniques

**Fig. 1:**
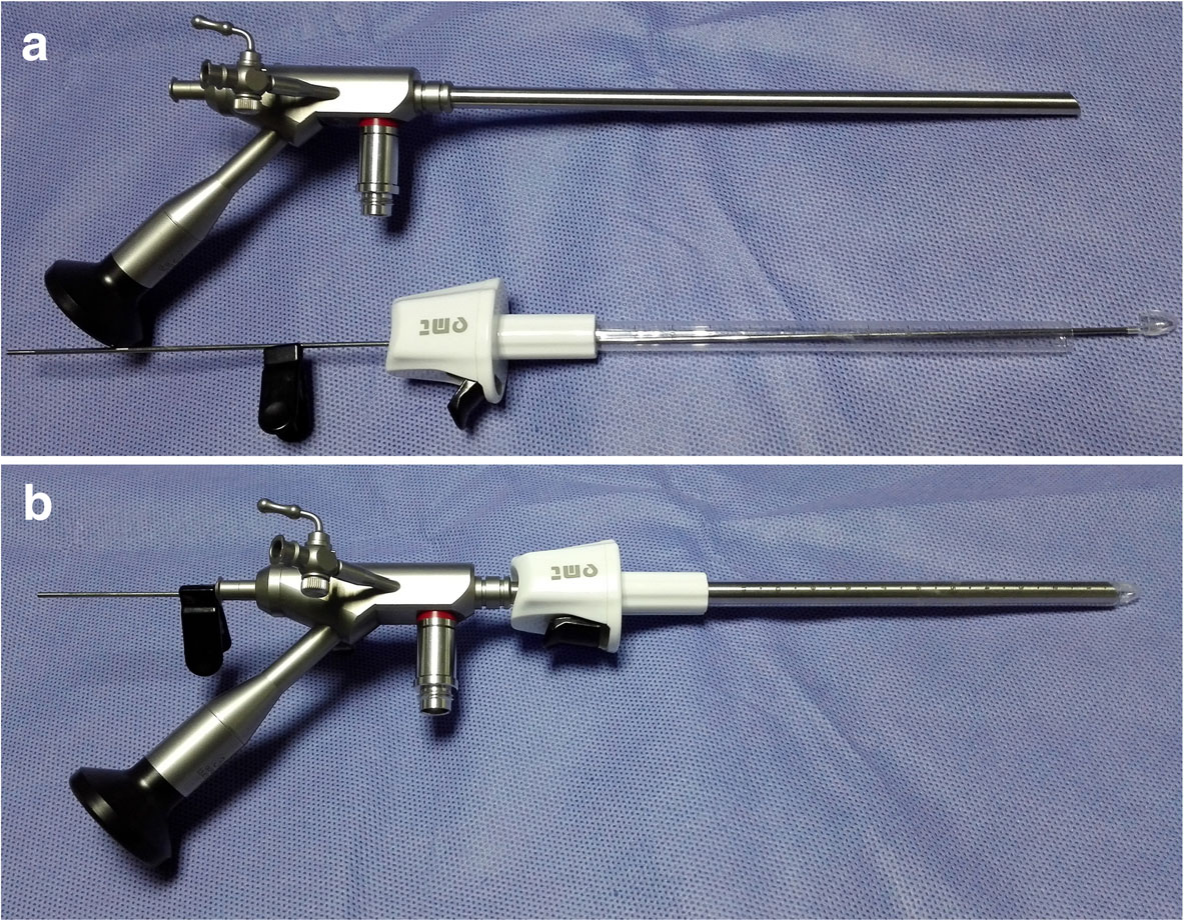
Transparent sheath and neuroendoscope before (**a**) and after (**b**) assembly

**Fig. 2:**
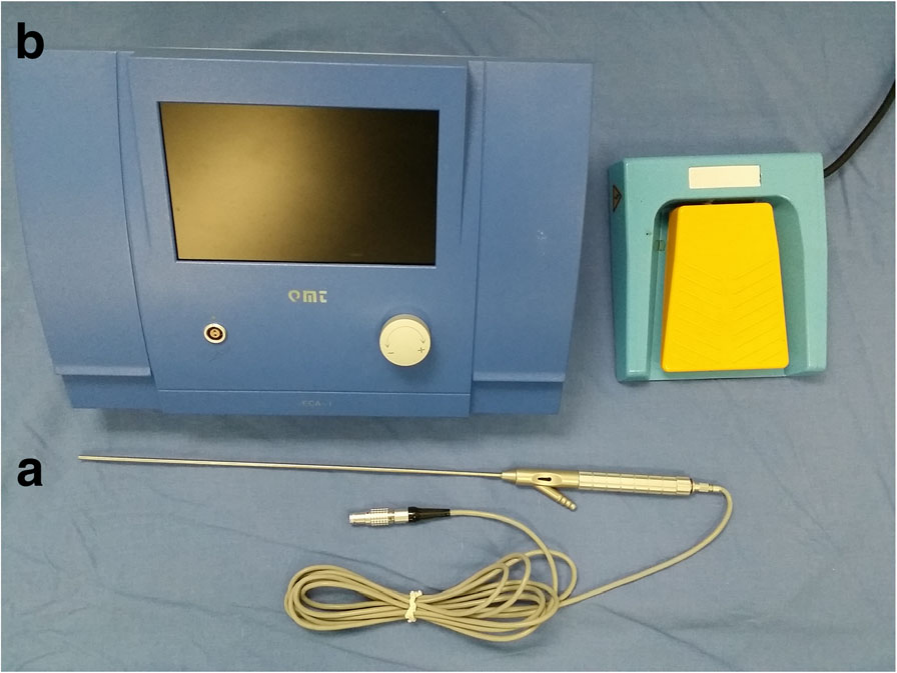
Hematoma smashing aspirator. **a** Smashing device (3.0-mm diameter). **b** Power system. The suction can be controlled by both a gear and a pedal

The INET equipment used in this study consisted of a high-definition imaging system, cold light source, Zeppelin large-working-channel endoscope, endoscope-dedicated bipolar coagulator (ZNE-242BIP, Zeppelin, Germany), and two of our patented inventions, namely, a transparent sheath (Chinese Patent No. ZL 200820046232.0) and a hematoma smashing aspirator (Chinese Patent No. ZL 201520248717.8). The Zeppelin large-working-channel endoscope (model number NEH/30-177-6.5) has a 177-mm working length, 6.5-mm body diameter, and 0° or 30° angle of view. The endoscope’s working channel diameter is 3.7 mm, and two 1.5-mm suction/flushing channels are integrated into the endoscope. The transparent sheath can be seamlessly fit with the neuroendoscope, its outer diameter is 7 mm, and it has an adjustable length. A transparent tip and a fixed device were also designed for the sheath. After a successful endoscope-guided puncture, the tip of the sheath can be removed together with the endoscope (Fig. 1). The diameter of the hematoma smashing aspirator, which contains a spiral suction device, is 3.0 mm, and it can be connected to a power system (Fig. 2).

**Fig. 3:**
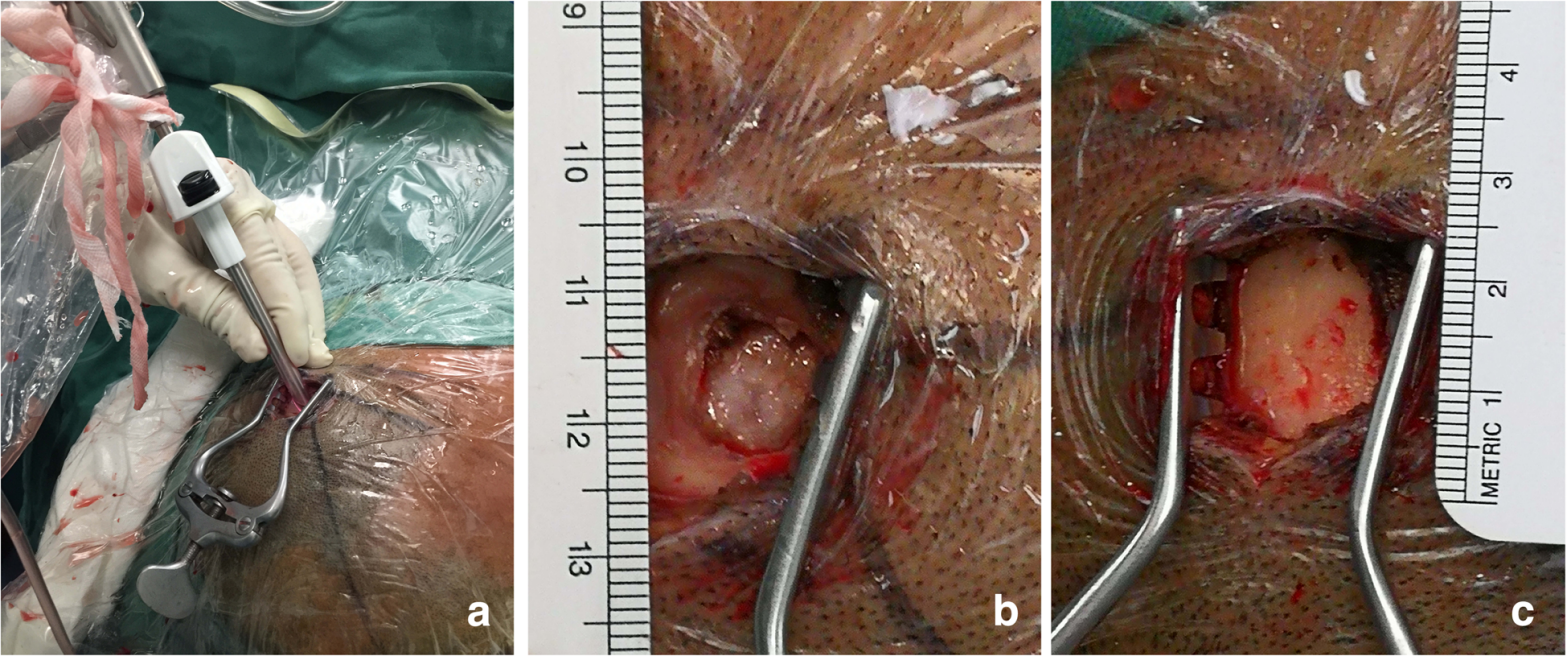
**a** Scalp incision (2.5–3.0 cm). **b** Bone hole (1 cm). **c** Endoscopic guided puncture

**Fig. 4:**
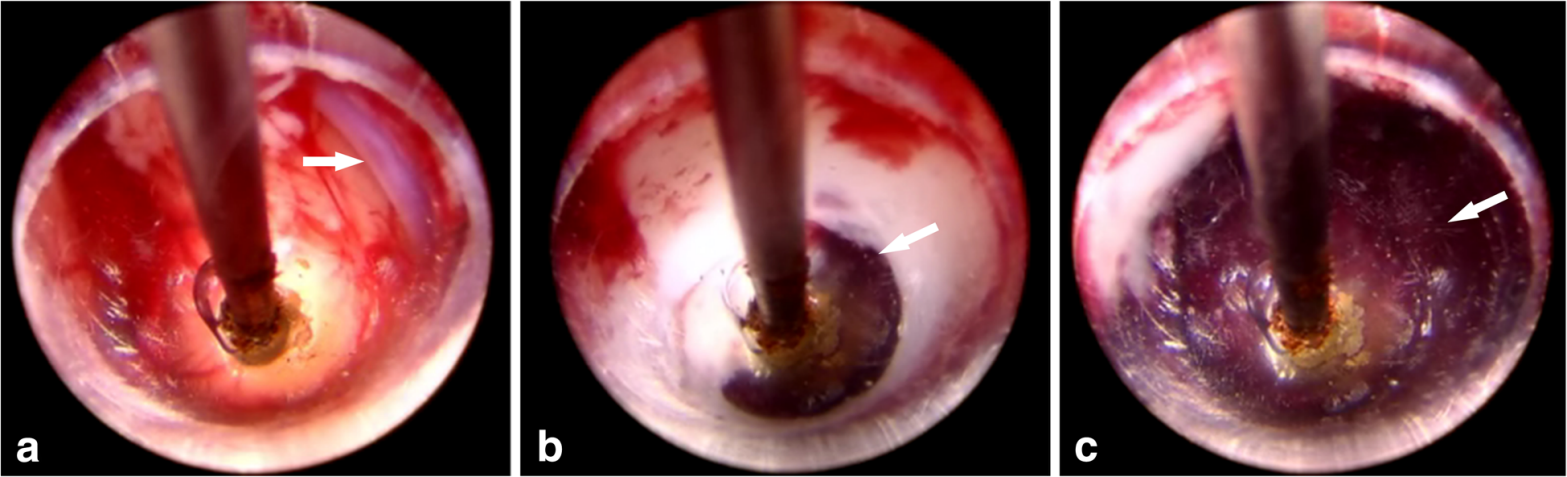
Visible hematoma puncture with INET. **a** Effective avoidance of a vessel (arrow) in front during puncture. **b** The endoscope carefully entered the edge of the brain parenchyma hematoma (arrow). **c** The endoscope completely entered the center of hematoma cavity (arrow)


The INET operation method for hematoma evacuation was performed as follows: the puncture point was selected according to the cerebral CT scan and was usually at the site where the hematomas had the shortest distance to the skin. Typically, the site was not close to large blood vessels and functional areas. We performed a 2.5–3 cm straight scalp incision and drilled a 1-cm bone hole, and the puncture process was performed visually (Fig. 3). During the puncture, the blood vessels in front could be avoided, and the margin and cavity of the hematoma were clearly visible (Fig. 4). After puncture, we removed the neuroendoscope together with the transparent sheath tip and placed the neuroendoscope again to evacuate the hematomas through the working channel. First, the most fragile hematomas were sucked and evacuated with the normal suction tube, and the hard hematomas were carefully cleared with the hematoma smashing suction apparatus. The suction force of the smashing suction apparatus could be manually controlled, and its inner axis could be adjusted to a shorter length than the sheath to help avoid vessel injury caused by improper suction. The small amount of bleeding stopped after the hematoma cavity was continuously washed with 39 °C saline solution. The active bleeding was stopped with an endoscopy-specific bipolar coagulation device. The clot was carefully removed under alternating conditions of air and water until the surrounding brain tissues were exposed. When the hematomas were removed, the brain tissue of the hematoma cavity wall could be seen through the transparent sheath (Additional file 1: Video 1). Finally, we carefully withdrew the sheath and routinely indwelled a drainage tube.

**Additional file 1: Video 1**. Brain tissue of the hematoma cavity wall is seen through the transparent sheath after removal of the hematoma. (WMV 1250 kb)


**Fig. 5:**
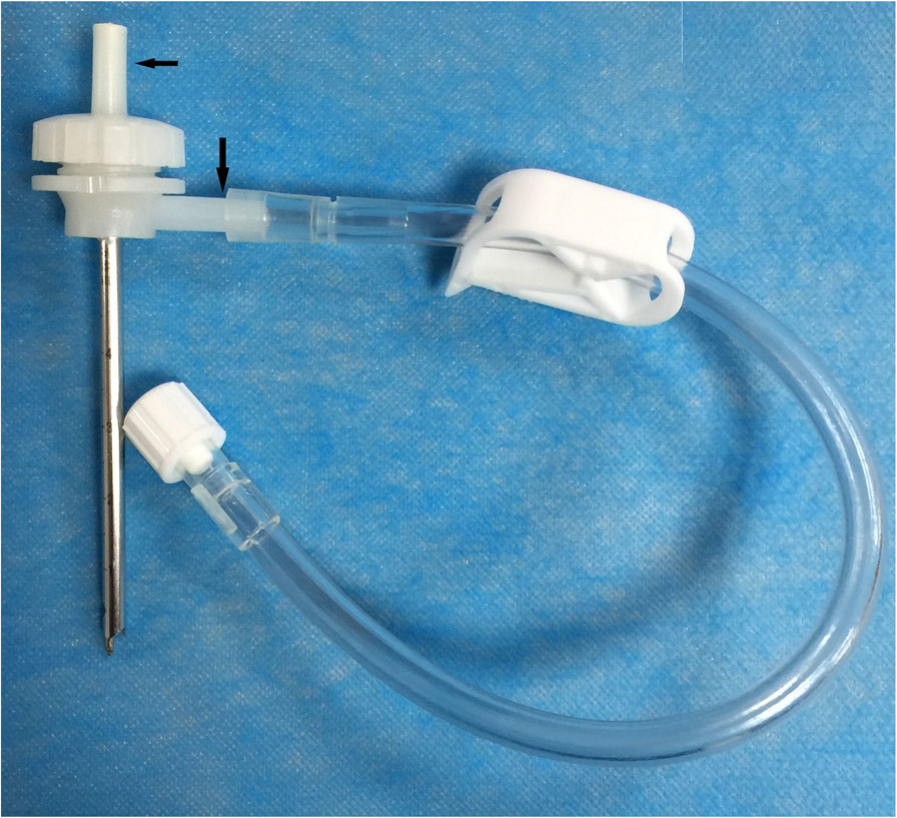
Puncture needle was connected to a drainage tube. Arrow denotes double channels

The CPDO method for hematoma evacuation was performed as follows: a set of hard channel puncture instruments (Wantfu Co. Ltd, Beijing, China, standard number YZB/State 1699-2014) was used to remove hematomas. The puncture needle consists of a double channel enclosed in a cannula with small holes at the end to allow infusion of lysis fluid (Fig. 5). The puncture site was based on CT scans of the patient and aimed to avoid functional domains and blood vessels. The puncture needle was fixed onto the skull after the needle point had been located in the center of the hematoma. As much of the hematoma as possible was aspirated. Lysis fluid was then injected under pressure to dissolve the residual hematoma to enable easy aspiration through the needle. The main component of the lysis fluid was urokinase (10,000–50,000 U based on the volume of hemorrhage) [25]. CT scanning was performed less than 12 h after the procedure to calculate the hematoma evacuation rate and again 1–3 days later to ascertain the position of the puncture needle and estimate the volume of any remaining blood. The drainage needle was retained in the brain for 3–5 days.

There was no difference in medical treatment between the two study groups. Hypertension was controlled early in the course of therapy if the systolic blood pressure was greater than 140 mmHg [26, 27].

### Outcome data

After surgery, the patients were sent to the intensive care unit (ICU) for medical treatment. CT scans were obtained less than 12 h postoperatively. The preoperative and postoperative hematoma volumes, hematoma evacuation rate, Glasgow Coma Scale (GCS) scores, Cerebral State Index (CSI), and complications (gastrointestinal stress ulcer bleeding, intracranial gas accumulation, intracranial infection, and cerebrospinal fluid leakage) were recorded 7 days after the operation. The follow-up surveys were performed 30 days after surgery. The 30-day mortality and Glasgow Outcome Scale (GOS) scores were collected from the patients’ medical records upon readmission, from the outpatient clinic records, or via telephone interview. GOS scores of 4–5 were defined as a good outcome, and GOS scores of 1–3 were defined as a bad outcome in this study.

### Statistics

The preoperative and postoperative hematoma volumes, operation time, preoperative GCS/CSI scores, and postoperative 7-day GCS/CSI scores were compared using an independent-sample *t* test. The 30-day mortality, hematoma evacuation rate, and complications were analyzed using the chi-squared test and Fisher’s exact test. Multivariable logistic regression model was constructed to analyze the association between the 30-day GOS and INET, age, and preoperative hematoma volumes. The data are reported as odds ratios (ORs) and 95% confidence intervals (CIs). Statistical analyses were performed using SPSS 13.0 software (SPSS Inc., Chicago, IL, USA). The threshold for statistical significance was set at *p* = 0.05.

## Results

### Clinical manifestations

A total of 98 patients were enrolled in this study, and the baseline indicators were compared between the two groups (Table 1). The proportions of patients under 65 years (*p* = 0.023) and with preoperative hematoma volumes less than 50 ml (*p* = 0.038) were significantly different between the two groups. The preoperative GCS scores and CSI showed no significant difference between the two groups. The patient bleeding sites or the major body of the hematoma were mainly located in the basal ganglia (75.4% in the INET group and 73.3% in the control group), and a few were distributed in the frontal lobe, temporal lobe, parietal lobe, and occipital lobe. There was no significant difference in the distribution of bleeding sites between the two groups.

### Short-term outcomes

The scalp incision and bone hole diameter were slightly larger in the INET group (Table 2). The operation time in the INET group was longer than that in the control group (65.2 ± 12.5 min versus 45.6 ± 10.9 min, *p* = 0.00), but the hematoma clearance rate was significantly higher (84 ± 7.1% versus 51 ± 8.4%, *p* = 0.00). The 7-day GCS scores (12.1 ± 1.65 versus 10.8 ± 1.5, *p* = 0.01) and CSI (88.7 ± 5.9 versus 80.1 ± 6.3, *p* = 0.02) were higher in the INET group.

**Table 2:** Comparison of intraoperation, postoperation, and 7-day follow-up indicators between the two groups

Indicators	INET group (*n* = 53)	Control group (*n* = 45)	*p* value
Scalp incision length (cm)	2.5–3.0	0.5–1.0	N/A
Skull drilling diameter (cm)	0.8–1.1	0.2–0.3	N/A
Operation time (min)	65.2 ± 12.5	45.6 ± 10.9	0.00
Evacuation rate (%)	84 ± 7.1%	51 ± 8.4%	0.00
7-day GCS	12.1 ± 1.6	10.8 ± 1.5	0.01
7-day CSI	88.7 ± 5.9	80.1 ± 6.3	0.02

### Medium-term outcomes and influencing factors

The 30-day mortality was lower (1.9% versus 15.6%, *p* = 0.036) in the INET group than in the control group. The postoperative complication rates of gastrointestinal stress ulcer bleeding (15.1% versus 24.4%, *p* = 0.243) and cerebrospinal fluid leakage (7.5% versus 4.4%, *p* = 0.829) showed no significant difference between the two groups. The incidence of intracranial gas accumulation after surgery was higher (77.4% versus 11.1%, *p* = 0.000), and the intracranial infection rate was lower (3.8% versus 20.0%, *p* = 0.011) in the INET group than in the control group (Table 3).

**Table 3:** Comparison of postoperative complication rates and mortality during 30 days in the two groups

Postoperative complication	INET group (*n* = 53)	Control group (*n* = 45)	*p* value
Gastrointestinal stress ulcer bleeding	8 (15.1%)	11 (24.4%)	0.243
Intracranial gas accumulation	41 (77.4%)	5 (11.1%)	0.000
Intracranial infection	2 (3.8%)	9 (20.0%)	0.011
Cerebrospinal fluid leakage	4 (7.5%)	2 (4.4%)	0.829
30-day mortality	1 (1.9%)	7 (15.6%)	0.036

Multivariable logistic regression analysis showed that a good medium-term outcome (GOS scores 4–5) was significantly associated with INET (OR 3.514, 95% CI 1.463–8.440, *p* = 0.005), age under 65 years (OR 1.402, 95% CI, 1.041–1.888, *p* = 0.026), and hematoma volume less than 50 ml (OR 1.974, 95% CI 1.302–2.993, *p* = 0.001) (Table 4).

**Table 4:** Effect of INET on brain parenchyma hematoma patients with good outcomes (GOS score of 4–5)

Variable	OR	95% CI	*p* value
INET applied (no)	Reference		
INET applied (yes)	3.514	1.463–8.440	0.005
Age ≥ 65 years	Reference		
Age < 65 years	1.402	1.041–1.888	0.026
Hematoma volume ≥ 50 ml	Reference		
Hematoma volume < 50	1.974	1.302–2.993	0.001

*OR* odds ratio, *CI* confidence interval. *p value of the Wald test

## Discussion

Neuroendoscopic techniques have progressed rapidly. The indication for operation has developed from the previous applications for intraventricular lesions and cystic lesions to those of brain parenchyma lesions. The endoscopic hematoma evacuation technique has been under development for nearly 20 years [9]. Several important technical developments were reported by various groups to enhance their orientation, visualization, and safety [9, 22]. One of the most important inventions was the transparent sheath that was used as a working channel for neuroendoscope and surgical instruments [14, 18]. In 2000, Nihishara first reported this improvement [18], and it was widely adopted by many surgeons for endoscopic hematoma evacuation surgery [13, 15, 20, 22, 28–30]. Compared with a steel sheath, the transparent sheath has the obvious advantage that the residual hematoma and hematoma-brain border can be easily identified through the transparent wall of the sheath (Additional file 1: Video 1). A deeper hematoma cavity can be clearly identified through the endoscope, which helps reduce bleeding and improve effectiveness and safety. This technique was first called neuroendoscopic control technology (NECT). In NECT, all the surgical instruments were used outside of the endoscope, and the main role of the endoscope was illumination. This surgical technique to remove the hematoma is effective, but several features, such as its non-visual implantation, larger sheath diameter, and difficulty in removing hard hematomas, still have room for improvement. In this study, we improved the transparent sheath and developed a hematoma smashing suction apparatus to achieve more minimally invasive removal of the hematoma.

### Summary of INET surgical capabilities

From this study, we can summarize the capabilities of INET as follows: (1) The puncture location was visualized, and the hematomas were evacuated from their center, as the vessels responsible for bleeding were mostly located around the hematoma cavity margin. (2) The brain tissue was white, and the hematomas were dark brown under endoscope. Most hematomas could be evacuated quickly in the air condition. If the boundary between the hematomas and brain tissue was not clearly identified, the hematoma cavity was washed using 39 °C saline and changed to the water condition to continue the evacuation. We found that real-time adjustment of the depth and angle of the transparent sheath in the hematoma cavity was secure. When we withdrew the sheath after the surgery, the bleeding sites in the puncture path were electrocoagulated. (3) After the operation, we routinely washed the hematoma cavity with 39 °C saline. Small amounts of bleeding were stopped with continuous washing. The active bleeding was stopped with endoscopy bipolar coagulation. (4) Removing the hematomas too quickly or forcefully evacuating clots adhering to vessels might lead to uncontrolled bleeding.

### Cerebral State Index (CSI)

CSI was obtained with a UP-8000 cerebral state monitor (the handset was provided by Danmeter AS Ltd, Denmark, and the mainframe was provided by the anesthesia depth lab of Shen-zhen Creative Industry Co., Ltd. The company was listed and registered in the Shenzhen Science and Technology Bureau). The instrument recorded spontaneous EEG through electrodes connected to the patient’s forehead, temple, and mastoid (behind ear) and quantified the patient’s level of consciousness and brain function through a fuzzy logic state analyzer. Our previous study [31] showed that CSI (0–100) could reflect the real-time coma depth and the coma dynamic process accurately; zero represented brain death, and 100 represented normal brain function. This study found that the whole brain function state showed obvious improvement after 7 days in the INET group (88.7 ± 5.9 versus 80.1 ± 6.3, *p* = 0.02).

### Comparison of the INET group and the control group for evacuation of brain parenchyma hematomas

The INET and CPDO methods both involve minimally invasive drilling techniques. In the present study, we safely achieved an average evacuation rate of 84 ± 7.1%, and the 30-day mortality rate was 1.9% using INET. The evacuation rate reported by other groups utilizing similar techniques (i.e., transparent sheath and endoscope) ranged from 80 to 99% [14, 16, 18, 28, 29], and the 30-day mortality rate ranged from 0 to 7.1% [8, 11–13, 20]. However, the hematoma evacuation rate in the control group was only 51 ± 8.4%, and its 30-day mortality was 15.6%. Because of the high hematoma clearance rates in the INET group, the hematoma mass effects and the hemotoxicity of the blood breakdown products were promptly relieved. Seven days after surgery, the average patient GCS scores and CSI were more improved than those of the control group.

The comparison of postoperative complications over 30 days in the two groups showed that the rate of intracranial gas accumulation was higher in the INET group (77.4% versus 11.1%, *p* = 0.000), and the gas accumulation occurred mainly in the forehead subdural and surgical area. Rinsing with saline after surgery reduced the surgical area gas accumulation. The forehead subdural gas accumulation occurred due to the volume contraction of brain tissue when using negative pressure to remove hematomas. One way to prevent air from entering was to use wet cotton sheets as padding around the bone hole. The incidence of intracranial infection in the control group was significantly higher than that in the INET group (3.8% versus 20.0%, *p* = 0.011), which was mainly due to the prolonged placement of the drainage tube and repeated injection of urokinase. The relatively longer operative time (65.2 ± 12.5 min versus 45.6 ± 10.9 min, *p* = 0.00) in the INET group than in the control group was not associated with an increased risk of cerebrospinal fluid leak (7.5% versus 4.4%, *p* = 0.829).

Multivariable logistic regression analysis showed that INET (OR 3.514, 95% CI 1.463–8.440, *p* = 0.005), age under 65 years (OR 1.402, 95% CI, 1.041–1.888, *p* = 0.026), and hematoma volume less than 50 ml (OR 1.974, 95% CI 1.302–2.993, *p* = 0.001) were independent predictors of good outcomes (GOS 4–5 scores) (Table 4). Therefore, INET surgery can provide a better outcome for patients with cerebral hemorrhage within 30 days, but determining the long-term prognosis of patients requires longer observations. Recently, Ma et al [23] used NECT to treat deep-seated basal ganglia hematomas and found that using their transparent sheath, which was different from ours, might have the potential to save patients and provide better long-term outcomes for patients with an ICH score of 3. Our transparent sheath was improved based on similar models; it was minimally invasive, and the hematoma removal efficiency of INET was similar.

### Limitations

The present study does have some limitations. First, it was a non-randomized concurrent control study conducted in a single center. Second, although strict inclusion/exclusion criteria were set and statistical methods were used to control selection bias, we still could not avoid the occurrence of it. Third, the surgeons responsible for surgery are trained doctors, but differences in individual clinical skills are unavoidable. Therefore, the conclusions require confirmation in future multicenter randomized controlled trial.

## Conclusions

For brain parenchyma hematomas, INET is a minimally invasive and safe surgical option. This modified technique with less complications might have the potential to save lives and provide better short- and medium-term outcomes for patients.

## Abbreviations

CI: Confidence interval
CPDO: Cranial puncture and drainage operation
CSI: Cerebral State Index
EHEO: Endoscopic hematoma evacuation operation
GCS: Glasgow Coma Scale
GOS: Glasgow Outcome Scale
ICH: Intracerebral hemorrhage
INET: Intra-neuroendoscopic technique
NECT: Neuroendoscopic control technology
OR: Odds ratio
